# The Intriguing Conundrum of a Nonconserved Multifunctional Protein of Citrus Tristeza Virus That Interacts with a Viral Long Non-Coding RNA

**DOI:** 10.3390/v13112129

**Published:** 2021-10-22

**Authors:** Sung-Hwan Kang, Vicken Aknadibossian, Laxmi Kharel, Shachinthaka D. Dissanayaka Mudiyanselage, Ying Wang, Svetlana Y. Folimonova

**Affiliations:** 1 Plant Pathology Department, University of Florida, Gainesville, FL 32611, USA; shk0015@auburn.edu (S.-H.K.); v.aknadibossian@ufl.edu (V.A.); 2 Department of Biological Sciences, Mississippi State University, Mississippi State, MS 39762, USA; 9laxsu9@gmail.com (L.K.); sdd292@msstate.edu (S.D.D.M.); wang@biology.msstate.edu (Y.W.)

**Keywords:** RNA virus, closterovirus, citrus tristeza virus, long non-coding RNA, plant virus, RNA-binding protein, RNA immunoprecipitation assay, electrophoretic mobility shift assay

## Abstract

*Citrus tristeza virus* (CTV), the largest non-segmented plant RNA virus, has several peculiar features, among which is the production of a 5′-terminal long non-coding RNA (lncRNA) termed low-molecular-weight tristeza 1 (LMT1). In this study, we found that p33, a unique viral protein that performs multiple functions in the virus infection cycle, specifically binds LMT1, both in vivo and in vitro. These results were obtained through the expression of p33 under the context of the wild type virus infection or along with a mutant CTV variant that does not produce LMT1 as well as via ectopic co-expression of p33 with LMT1 in *Nicotiana benthamiana* leaves followed by RNA immunoprecipitation and rapid amplification of cDNA ends assays. Further experiments in which a recombinant p33 protein and an in vitro transcribed full-length LMT1 RNA or its truncated fragments were subjected to an electrophoretic mobility shift assay demonstrated that p33 binds to at least two distinct regions within LMT1. To the best of our knowledge, this is the first report of a plant virus protein binding to a lncRNA produced by the same virus. The biological significance of the interaction between these two viral factors is discussed.

## 1. Introduction

*Citrus tristeza virus* (CTV), a member of the family *Closteroviridae*, is the largest non-segmented plant RNA virus that is responsible for two of the most destructive viral diseases of citrus: quick decline and stem pitting [1,2,3,4,5,6,7,8]. The 19.3 kb single-stranded positive-sense RNA genome of CTV is encapsidated by two coat proteins, the major and minor coat proteins (CP and CPm, respectively), resulting in a long flexuous virion (2000 nm by 10–12 nm). Apart from the genomic RNA (gRNA) and its negative-sense complimentary copy, CTV produces more than 30 sub-genomic RNAs (sgRNAs) and a number of different defective RNAs upon replication in the infected cells [9,10,11,12]. Among those are the two positive-sense 5′-coterminal non-coding sgRNAs termed low-molecular-weight tristeza 1 (LMT1) and LMT2 [11,13]. LMT2 (~650 nucleotides (nts)) has no known function and is thought to be processed during virion assembly [14]. On the other hand, LMT1 (~750 nts) is generated through replication by initiation at the 3′ end of the negative strand copy of gRNA and termination at the respective controller element [11,15]. Recently, we showed that LMT1 plays a role in counter-acting the host defense responses and is required for citrus infection [16].

**Figure 1:**
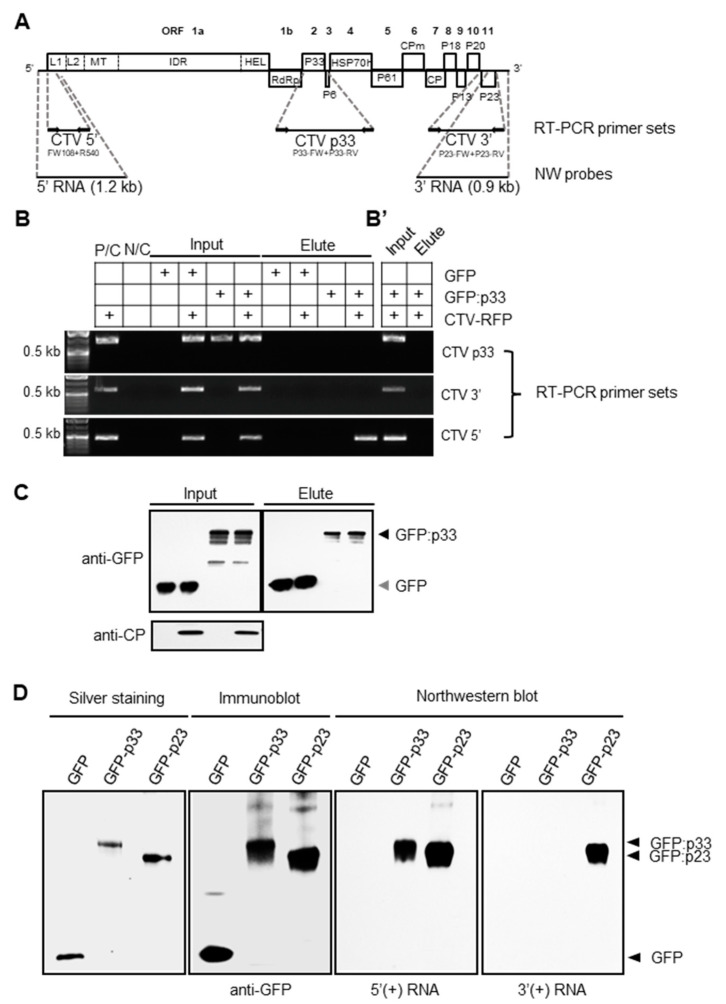
RNA-binding property of the p33 protein. (**A**) Schematic representation of the CTV genome and the regions targeted for the amplification and probe hybridization of RNA bound to the protein trapped via anti-GFP antibody-conjugated agarose beads in the RIP assay. The boxes represent ORFs and their translated products. L1 and L2, papain-like leader proteases; MT, methyltransferase; IDR, large interdomain region; HEL, helicase; RdRp, RNA-dependent RNA polymerase; CPm, minor coat protein; HSP70h, heat shock protein 70 homolog; CP, coat protein. Sequences of primers can be found in Supplementary Table S1. (**B**) RT-PCR analysis of the RNA extracted at two steps (“Input” and “Elute”) of the RIP assay. Input:—the supernatant of the leaf homogenate in the lysis buffer after centrifugation at 3000× *g*; Elute:—beads left at the final step of RIP after repeated washes. The table above the agarose gel image shows the components expressed in leaves of *N. benthamiana* in the RIP assay treatments. P/C, positive control in which leaves were infiltrated with pCTV:RFP; N/C, negative control in which leaves were infiltrated with the empty vector. The agarose gel images show products amplified by RT-PCR using the set of primers targeting the p33 ORF, the 3′ terminus, or the 5′ terminus of the CTV genome (top, middle, and bottom, respectively) as shown in (**A**). (**B’**) RT-PCR analysis of the RNA extracted at two steps (“Input” and “Elute”) of the RIP assay as described in (**B**), except that the RT step was performed using forward primers to detect negative-stranded RNA by subsequent PCR. (**C**) Immunoblot analysis of the total protein extracts from the supernatant of the homogenate and the beads at the final step of RIP assay (“Input” and “Elute”, respectively; see Section 2). Input represented all the components expressed in *N. benthamiana* using agroinfiltration. Elute contained trapped GFP-tagged p33 or GFP. (**D**) GFP and GFP-tagged CTV proteins (GFP:p33 and GFP:p23) expressed individually in *N. benthamiana* were purified using GFP-Trap and analyzed by SDS-PAGE. “Silver staining” shows the proteins separated by SDS-PAGE, “Immunoblot” shows detection of GFP, GFP:p33 and GFP:p23 using anti-GFP antibody. “Northwestern blot”: proteins transferred from the gel onto the nitrocellulose membrane were renatured and subsequently incubated with in vitro-transcribed DIG-labelled RNAs (5′ and 3′ probes targeting the regions indicated in (**A**)). Both RNA probes (5′ (+) and 3′(+) RNAs) were bound to GFP:p23 known as non-specific RNA binding protein, however, only 5′(+) RNA probe was bound to GFP:p33.

The CTV genome encodes 12 open reading frames (ORFs; Figure 1) [9,17]. ORFs 1a and 1b are translated directly from gRNA and code for polyproteins that function in virus replication [3,9,18]. The remaining ten ORFs are expressed from 3′-coterminal sgRNAs. The CP, CPm, p65, and p61 proteins are required for virion assembly and, along with p6, for virus movement [3,19,20]. CP, p20, and p23 act as viral suppressors of host RNA silencing (VSRs) [21]. CTV also produces three unique proteins—p13, p18, and p33—which extend the virus host range [20,22].

The nonconserved p33 protein was shown to perform multiple functions in the CTV infection cycle. It plays a crucial role in virus superinfection exclusion by mediating the ability of a variant of CTV to exclude superinfection by a closely related virus variant [23,24]. Recently, it was also demonstrated that p33 is a viral effector that modulates the host immune response and, thus, influences the virus’s pathogenicity [25]. Furthermore, p33 has been implicated in virus translocation throughout the host plants. Although p33 is not essential for systemic infection in a number of citrus hosts, the protein influences the efficiency of the virus systemic spread [22,26,27]. Moreover, it is indispensable for CTV infection of certain other hosts (e.g., sour orange and lemon) [22]. The protein itself was shown to possess multiple characteristics of viral movement proteins (MPs). In particular, p33 has the ability to form extracellular tubules, which appear to originate from the deposits of p33 at the plasmodesmata [26,28]. The MPs of other tubule-forming viruses such as like *Cowpea mosaic virus* and *Grapevine fanleaf virus* have been reported to interact with their respective CPs, and this interaction was required for virion transport [29]. Similarly, the CTV p33 also interacts with the viral CP [30]. All these observations led to the suggestion that p33 could play a role of a noncanonical MP, which is needed for virus translocation in the selective hosts.

To date, at least 36 plant virus MPs were reported to bind nucleic acids, all in a sequence-non-specific manner [31]. For instance, the ability of *Red clover necrotic mosaic virus* (RCNMV) and *Prunus necrotic ringspot virus* MPs to bind RNA was required for cell-to-cell movement [32,33], while that of a *Poa semilatent virus* MP, TGBp1, was needed for virus systemic movement [34]. Ultimately, the RNA-binding property of viral MPs seems to play an integral part in virus movement. In this work, we show that the p33 protein of CTV is an RNA-binding protein. However, unlike many other MPs of plant viruses, which bind RNA in a sequence-non-specific manner, p33 binds a particular RNA species produced during virus replication in the infected cells—a viral long non-coding RNA (lncRNA) LMT1. The p33 protein interacts with LMT1 both in vivo and in vitro, and the interaction is in a cooperative manner, suggesting multiple binding sites in the LMT1 sequence.

## 2. Materials and Methods

### 2.1. In-Silico Sequence Analysis

The prediction of RNA binding motif was performed with Genesilico Fold Prediction Metaserver (https://www.genesilico.pl/meta2/, accessed on 6 October 2011) and InterPro (http://www.ebi.ac.uk/interpro/, accessed on 6 October 2011) using the p33 amino acid (aa) sequence of the T36 isolate of CTV (EU937521).

### 2.2. Agroinfiltration into Nicotiana Benthamiana

Agroinfiltration of constructs was conducted as previously described [26]. Briefly, plasmids were introduced by heat shock into *Agrobacterium tumefaciens* EHA105, and the resulting transformants were selected on Luria-Bertani agar plates containing two antibiotics (50 µg/mL rifampicin and 25 μg/mL kanamycin). Cells collected from an over-night culture of a selected single colony were gently resuspended in a buffer containing 10 mM 2-(N-morpholino) ethane sulfonic acid (MES, pH 5.85), 10 mM MgCl_2_ and 150 mM acetosyringone at O.D._600nm_ = 1.0. After a 3 h incubation at room temperature without shaking, the suspension was infiltrated into six-week-old *N. benthamiana* plants using a needleless syringe.

### 2.3. RNA Immunoprecipitation Assay

Total protein was extracted from the infiltrated leaves of *N. benthamiana* by grinding in a lysis buffer (50 mM Tris-HCl, 1 mM EDTA, 150 mM NaCl, 5% glycerol) containing protease inhibitor cocktail (AEBSF, Bestatin, E-64, Leupeptin, Pepstatin A, and 1,10-Phenanthroline; MilliporeSigma, Burlington, MA, USA). The homogenate was incubated on ice for 1 h with gentle shaking on the rocker and then clarified by centrifugation at 3000× *g* for 10 min at 4 °C. The supernatant (600 μL) was immunoprecipitated using GFP-Trap^®^ or RFP-Trap^®^ (ChromoTek GmbH, Planegg-Martinsried, Germany)—anti-green fluorescent protein (GFP) or anti-red fluorescent protein (RFP) antibody-conjugated agarose beads (50 µL)—by incubating for 2 h on ice with gentle shaking. The supernatant described at this step was also used as an ‘Input’ sample for reverse transcription-PCR (RT-PCR) or immunoblot analysis. The binding reaction was washed three times as per the manufacturer’s instruction by centrifugation at 3000× *g* for 2 min at 4°C. The pellet of the agarose beads collected at this step was used as ‘Elute’ for further analysis. For the immunoblot analysis, total protein was extracted as described below. For RT-PCR analysis, total RNA was extracted using the TRIzol™ reagent (Invitrogen, Waltham, MA, USA) according to the manufacturer’s procedure. RNA extracts were resuspended in 50 μL of RNase-free water.

### 2.4. RT-PCR Assay

Total RNA extracts described above were measured using NanoDrop UV-Vis Spectrophotometer (Thermo-Fisher Scientific, Waltham, MA, USA), diluted with RNase-free water to adjust concentration to 1 ng/µL, and used as a template for an RT-PCR reaction. RT-PCR was performed using SuperScript™ III One-Step RT-PCR System with Platinum Taq DNA Polymerase (Invitrogen, Waltham, MA, USA) in 20 µL volume (10 µL 2X reaction buffer, 1 µL template RNA, 0.5 µL forward primer of 20 µM, 0.5 µL reverse primer of 20 µM, 1 µL enzyme mix, and nano-pure water up to 20 µL). Complementary DNA (cDNA) synthesis was followed immediately by PCR amplification according to the manufacturer’s procedure with 30 cycles. Reaction products were analyzed by electrophoresis in 1% agarose gels containing ethidium bromide at 200 ng/mL.

### 2.5. Immunoblot Analysis

All fractions were mixed with the equal volume of the 2X sample loading buffer (125 mM Tris-HCl, 4% (*w*/*v*) SDS, 20% (*v*/*v*) glycerol, and 0.01% (*w*/*v*) bromophenol blue) with dithiothreitol and boiled for 10 min. Mixtures were briefly centrifuged prior to loading, electrophoresed through 10% SDS-polyacrylamide gels, and electro-transferred to a polyvinylidene difluoride (PVDF) membrane. The membrane was blocked with 1X TBS-T (137 mM NaCl, 27 mM KCl, 250 mM Tris-HCl, and 0.1% Tween-20) containing 5 % (*w*/*v*) skim milk for an hour at room temperature prior to incubating with an anti-GFP, anti-RFP (dilution: 1:1000; Santa Cruz Biotechnology, Dallas, TX, USA) or anti-CP antibody (CTV-908 [35]; dilution: 1:500) with 1X TBS-T for another hour. Anti-rabbit IgG conjugated to horseradish peroxidase was used as a secondary antibody (dilution: 1:20,000; Santa Cruz Biotechnology, Dallas, TX, USA), and the signal was visualized on chemiluminescence film (X-OMAT LS; Carestream Health Rochester, NY, USA) in a dark room (Carestream Kodak, Sigma-Aldrich, St. Louis, MO, USA).

### 2.6. Determination of the 5′ and 3′ Ends of RNA by Rapid Amplification of cDNA Ends (RACE)

The RNA isolated from the agarose beads at the “Elute” step of the RNA immunoprecipitation (RIP) assay was used for the amplification and the RACE assays (SMARTer RACE kit; Clontech, Takara Bio Inc., Kusatsu, Japan) to determine the 5′ and 3′ ends of co-immunoprecipitated RNA. To determine the 5′ end, a random primer and manufacturer-supplied adapter oligos were used for the first-strand cDNA synthesis. The 5′ RACE product was amplified using the forward primer supplied by the manufacturer and a CTV-specific reverse primer (GSP1). To determine the 3′ end of the RNA, the isolated RNA was polyadenylated using *Escherichia coli* poly (A) polymerase (Applied Biosystems, Waltham, MA, USA). The first strand synthesis and 3′ RACE was performed as instructed (SMARTer RACE kit; Clontech, Takara Bio Inc., Kusatsu, Japan) with a manufacturer-supplied reverse primer and a CTV-specific forward primer (GSP2). Both 5′ and 3′ RACE products were cloned into the manufacturer-supplied vector and selected clones (10 for 5′ RACE and 100 for 3′ RACE) were sequenced (Psomagen, Rockville, MD, USA).

### 2.7. Northwestern Blot Analysis

Protein samples (approximately, 2 µg) were separated by electrophoresis through 10% SDS-PAGE and transferred onto a nitrocellulose membrane using semi-dry Trans-Blot apparatus (Bio-Rad Laboratories, Hercules, CA, USA). The membrane was incubated at room temperature in the RN (renaturation) buffer (10 mM Tris-HCl (pH 7.5), 1 mM EDTA, 50 mM NaCl, 0.1% Triton X-100 and 1X Denhardt’s reagent [36]) for 20 min. The incubation was repeated three times. Then, the membrane was incubated with a digoxigenin (DIG)-labeled riboprobe specific to the positive-strand RNA corresponding to the 5′ or 3′ end of the CTV genome generated as described in Satyanarayana et al. [37], which was generated using a DIG-RNA labeling kit as per the manufacturer’s instruction (Roche, Basel, Switzerland) in the RN buffer for one hour with slight agitation. The membrane was cross-linked by UV (auto-crosslink, Stratagene, San Diego, CA, USA). The membrane was washed three times for 20 min each with the RN buffer without Triton X-100 and Denhardt’s reagent prior to the blocking in 1X maleate buffer. The membrane was treated with an anti-DIG antibody (anti-DIG-AP, Fab fragments, Roche, Basel, Switzerland) and detection was carried out with the alkaline phosphatase chemiluminescent substrate (CSPD; Roche, Basel, Switzerland) as described in the manufacturer’s instructions (Roche, Basel, Switzerland).

### 2.8. Protein Expression and Purification

The p33 or the transmembrane domain (TMD) lacking p33 (p33ΔTMD) coding sequences were amplified using P33-FW and either P33-RV or P33ΔTMD-RV (Supplementary Table S1) and were ligated into the glutathione S-transferase (GST) fusion protein expression vector pGEX-4T-1 (Cytiva, Marlborough, MA, USA) between *EcoR* I and *Not* I restriction enzyme recognition sites. The constructs were transformed into Rosetta strain (DE3) (MilliporeSigma, Burlington, MA, USA) competent cells. Protein expression induction was performed at OD_600 nm_ 0.5–0.7 with 0.4 mM IPTG, and the cultures were then incubated at 20 °C for overnight. Pellets were resuspended in 1X PBS buffer (137 mM NaCl, 2.7 mM KCl, 10 mM Na_2_HPO_4_, and 1.8 mM KH_2_PO_4_) supplemented with 20 mM PMSF and sonicated to lyse the cells. The homogeneous cell lysate was centrifuged at 10,000× *g* for 30 min at 4 °C. The supernatant was collected and incubated for 1 h with 2 mL of 50% slurry of Glutathione Resin (GenScript, Piscataway, NJ, USA) before loading onto an empty EconoPac gravity-flow column (Bio-Rad Laboratories, Hercules, CA, USA). The resin was then washed with 10 mL 1X BS followed by elution with 10 mL elution buffer (50 mM Tris-HCl at pH 8.0 and 10 mM reduced glutathione). The elutes were concentrated using an Amicon protein concentrator (MilliporeSigma, Burlington, MA, USA). Proteins were then separated by 8% SDS-PAGE electrophoresis followed by Coomassie blue staining and destaining to estimate concentration using a BSA standard as reference. Purified GST:p33 and GST elutes were run on 12% Mini-PROTEAN^®^ TGX™ Precast Gels (Bio-Rad Laboratories, Hercules, CA, USA) and electro-transferred to PVDF membrane. Western blot was carried out with 0.5 µg/mL anti-GST tag rabbit anti-tag polyclonal primary antibody (Invitrogen, Waltham, MA, USA) and 1:20,000 goat anti-rabbit IgG (H + L) (HRP) secondary antibody (Abcam, Cambridge, UK) after blocking with 5% non-fat milk in TBST. The PVDF membrane was treated with Clarity™ Western ECL Substrate (Bio-Rad Laboratories, Hercules, CA, USA) and visualized on Bio-Rad ChemiDoc™ XRS+ system with Image Lab™ software.

### 2.9. Construction of Clones Encoding LMT1 and LMT1 Truncations for In Vitro Transcription

A series of LMT1 clones was constructed. A fragment encompassing an extended full-length LMT1 (nt positions 1 to 780 in the CTV genome) and its truncated versions encompassing nt positions 201–400, 401–600, and 601–780 (fragments A, B, and C, respectively) were amplified by PCR using set of primers provided in Supplementary Table S1 (primer names begin with LMT). The amplified products were directly cloned into pGEM^®^-T Easy plasmid (Promega, Madison, WI, USA) and sequenced using a pair of M13 primers listed in Supplementary Table S1 (Psomagen, Rockville, MD, USA) to verify the orientation of the insertions. For in vitro transcription, the plasmids harboring fragments A and B were linearized by *Nde* I (NEB, Ipswich, MA, USA) prior to transcription using T7 MEGAscript™ kit (Thermo-Fisher Scientific, Waltham, MA, USA). Plasmids harboring the full-length LMT1 and fragment C were linearized by *Nco* I (NEB, Ipswich, MA, USA) before transcription using the SP6 MEGAScript™ Kit (Thermo-Fisher Scientific, Waltham, MA, USA).

### 2.10. Electrophoretic Mobility Shift Assay (EMSA)

The indicated amounts of purified GST:p33ΔTMD or GST were incubated with 1 µM of an RNA transcript in 10 µL binding buffer (10 mM HEPES-NaOH pH 8.0, 50 mM KCl, 100 mM EDTA, and 5% glycerol) for 20 min at 28 °C with gentle shaking. Subsequently, the incubated mixture was electrophoresed in 6% non-denaturing PAGE using 1X TAE buffer and transferred onto Hybond™-XL nylon membrane (Amersham Biosciences, Little Chalfont, United Kingdom) via a semi-dry transfer cassette (Bio-Rad Laboratories, Hercules, CA, USA) and was immobilized by a UV-crosslinker (UVP, Upland, CA, USA). The crosslinked membrane was blocked with Denhardt’s solution (VWR, Radnor, PA, USA) for 30 min at 60 °C before adding a DIG-labeled probe. After overnight hybridization, the location of RNA transcripts was detected using DIG-Northern-Detection-system (Roche, Basel, Switzerland) per the manufacturer’s instructions. The Signals were captured by a ChemiDoc™ (Bio-Rad Laboratories, Hercules, CA, USA). The Hill equation was plotted using the default function in Prism version 8 (GraphPad, San Diego, CA, USA).

## 3. Results

### 3.1. The p33 Protein Is A Sequence-Specific RNA-Binding Protein

In-silico analysis of the aa sequence of the p33 protein from the T36 isolate of CTV predicted a canonical arginine-rich RNA-binding motif (ARM) in the N-terminal region of the protein (Supplementary Figure S1; see Section 2), which implied the ability of p33 to bind RNA molecules via a well-conserved ARM (reviewed in [38]). To examine whether p33 binds viral RNA produced during CTV infection, we first used a RIP approach. The p33 protein tagged with the GFP was co-expressed with CTV in *N. benthamiana* plants through infiltration with an *A. tumefaciens* culture transformed with a binary vector carrying a fusion of the p33 ORF with that of GFP (pGFP:p33; [26]) positioned under the *Cauliflower mosaic virus* 35S promoter along with a culture transformed with a vector carrying a cDNA clone of CTV tagged with an RFP gene (pCTV-RFP; [35]). At five days post infiltration (dpi), expression of GFP:p33 and CTV-RFP in the infiltrated leaves was confirmed by observation of GFP and RFP fluorescence, respectively (data not shown). Tissue extracts obtained from the infiltrated leaves were subjected to RIP using anti-GFP antibody-linked beads (GFP-Trap^®^). To analyze the RNA bound to the GFP-tagged p33 protein, total RNA extracted from the beads at the final “Elute” step (see Section 2) was used as a template for RT-PCR with primer sets corresponding to three different regions of the CTV genome (Figure 1A). All primer sets successfully amplified the corresponding target regions from a total RNA extract of the leaves infiltrated solely with pCTV-RFP, which was used as a positive control for RT-PCR, as well as from the extracts of leaves co-expressing CTV-RFP and GFP:p33 that were obtained prior to the RIP procedure (Figure 1B; P/C and “Input”, respectively). No amplification was detected from the total RNA extract from the leaves of the control plants infiltrated with an empty binary vector (Figure 1B; N/C). In contrast, RT-PCR using post-RIP GFP-p33-bound RNA obtained via co-immunoprecipitation of extracts from GFP:p33- and CTV-RFP-bearing samples resulted in the amplification of only the fragment corresponding to the 5′-proximal region of the CTV genome (Figure 1B; “Elute”). No amplification of the other two fragments corresponding to the central or the 3′-terminal genomic regions was detected. The RIP-subjected samples from the control plants expressing GFP and CTV-RFP did not yield any amplification, indicating that there was no binding of the CTV RNA to GFP. Presence of the protein components such as GFP:p33, GFP, and the viral CP in the respective fractions was verified by immunoblotting using a GFP-specific antibody for GFP and GFP:p33 and a CTV CP-specific antibody for CTV-RFP (Figure 1C).

The results of the above experiment suggested that p33 could bind a specific RNA, which sequence corresponds to that of the 5′ region of the virus genome. As CTV generates both negative- and positive-stranded RNAs during replication [8,10], the polarity of the CTV RNA bound to GFP:p33 needed to be examined (Figure 1B’). Accordingly, we attempted to synthesize a cDNA from an RT reaction using the primers intended to anneal only to the negative-stranded copy of CTV RNA at positions located at the 3′, central, and 5′ regions, which was followed by PCR (Figure 1A,B’). As a result, no amplification of the fragments corresponding to any of the three regions was detected when the GFP:p33-bound RNA was used as the template, suggesting the absence of negative-stranded CTV RNAs.

To validate the results obtained by RIP and further characterize RNA binding properties of p33, we employed a different experimental technique—the northwestern blot analysis—and included another protein of CTV, p23, which is known as a non-sequence-specific RNA binding protein, as a control [39]. The GFP-tagged p33 and p23 proteins ([26,30], respectively) along with free GFP were individually expressed in the *N. benthamiana* leaves as described above and purified using GFP-Trap^®^. The purified proteins were verified via SDS-PAGE followed by silver staining and immunoblotting using the GFP-specific antibody. The northwestern blot analysis using DIG-labelled RNA probes comprising the 5′- or 3′-terminal ~1 kb-region of the viral gRNA (Figure 1A) showed that both 5′ and 3′ probes were bound by the p23 protein, which was in the agreement with the previous finding that this protein of CTV binds RNA in a non-sequence-specific manner (Figure 1D). On the other hand, the p33 protein was found to bind only the 5′-terminal RNA probe that comprised the 5′-terminal 1.2-kb of gRNA but not the probe containing the 3′-terminal 0.9-kb region of CTV gRNA. No binding between free GFP and either probe was detected. The obtained results indicated that the p33 protein is an RNA-binding protein and suggested that p33 could bind a specific RNA molecule(s) produced during virus infection.

### 3.2. p33 Binds A Single sgRNA Species Produced from the 5′ End of the CTV Genome

**Figure 2:**
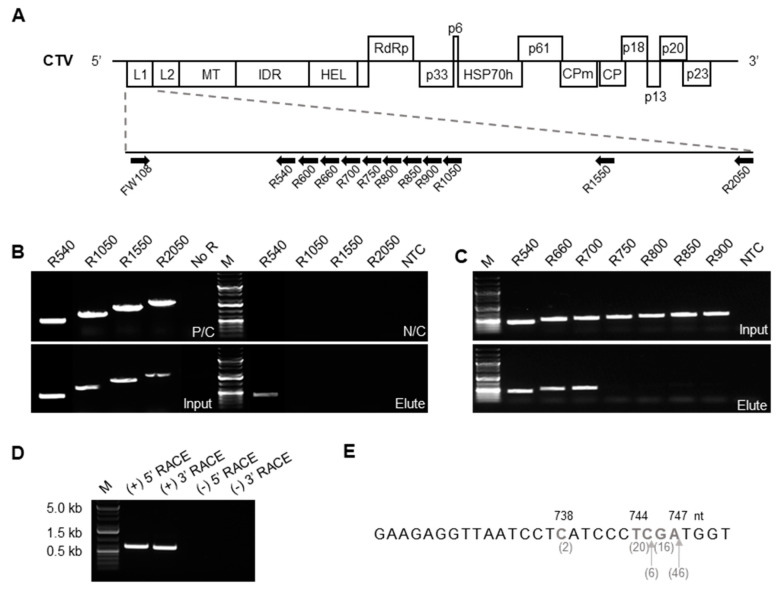
RNA molecule bound to the p33 protein is produced from the 5′ end of the CTV genome. (**A**) Schematic representation of the CTV genome and the positions of primers used in the RT-PCR analysis (shown in (**B**,**C**)). FW108 is a forward primer that corresponds to the nt position 108 in the CTV genome. Reverse primers (R) are named according to the nt positions they bind to the CTV genome (see Supplementary Table S1). (**B**) RT-PCR products of primer sets shown in (**A**) were analyzed by agarose gel electrophoresis. Molecular weight marker (M) has three bright bands indicating fragments of 0.5, 1.5, and 5.0 kb from the bottom to the top, respectively (as shown in (**D**)). P/C, RT-PCR using total RNA extract from CTV-infected tissue as a template. N/C, RT-PCR using water template. Input, see Figure 1B. Elute, total RNA extracted from the GFP-Trap beads at the completion of RIP assay (see Section 2). (**C**) RT-PCR products of primer sets shown in (**A**) were analyzed by agarose gel electrophoresis (continued from (**B**)). The image shows that the amplification no longer occurs with reverse primers annealing beyond 750 nt position of the CTV genome. “Input” and “Elute” are described in (**B**). (**D**) 5′ and 3′ RACE for positive- and negative-stranded RNA using “Elute” RNA analyzed by agarose gel electrophoresis. Each amplified product shown in (+)5′ RACE and (+)3′ RACE lanes was excised from the gel and cloned for sequencing. RACE reaction targeting negative-strands ((-)5′ RACE and (-)3′RACE) produced no amplification. (**E**) The 3′ end sequence of (+)3′RACE products determined by sequencing is shown. 46 out of 90 clean reads (out of 100 clones) ended at 747 nt position of the CTV genome. 16, 6, 20, and 2 reads ended at 746, 745, 744, and 738 nt positions, respectively. NTC, no template control.

Upon replication, CTV produces more than 30 species of sgRNA molecules, including a set of different 5′ co-terminal sgRNAs [8]. Based on the results of our experiments discussed above, we speculated that p33 binds one of the latter RNAs. In order to identify which RNA was bound to p33, we first attempted to determine its size by performing RT-PCR using a forward primer corresponding to the 5′-terminal region of the CTV genome (FW108) and a series of reverse complementary primers annealing to variable positions within the first 2050 nts in the 5′ genomic region (Figure 2A). The analysis was done in two steps: four different reverse primers binding at positions corresponding to nts 540, 1050, 1550, and 2050 in the CTV genome (R540, R1050, R1550, and R2050, respectively; see Supplementary Table S1) were used in the initial step, while additional reverse primers (see below) were used in the second step to narrow down the size of the RNA molecule bound to p33. All primer sets successfully amplified the target regions from the positive control—a total RNA extract from leaves infiltrated with pCTV-RFP—and from the “input” samples bearing CTV-RFP and GFP:p33, confirming that the designed primer sets can efficiently detect the respective sequences in the CTV genome (Figure 2B,C). In contrast, the initial assessment using post-RIP GFP:p33-bound RNA demonstrated that among the four reverse primers only R540 yielded the amplification of a respective product, while no amplification was detected with the other three primers (Figure 2B). This suggested that an RNA molecule(s) in the “Elute” fraction was shorter than 1050 nts. To narrow down the size of the RNA molecule(s), we conducted an RT-PCR analysis using a series of six additional reverse primers (R660, R700, R750, R800, R850, R900) annealing at six different positions upstream of 1050 nt position (Figure 2C). Among those, RT-PCR using only R660 and R700 yielded amplification of the corresponding products, which suggested that the template RNA molecule(s) were longer than 700 but shorter than 750 nts.

Subsequently, 5′ and 3′ RACE assays were employed to further elucidate the precise size of the RNA found in the association with the p33 protein. Each 5′ or 3′ RACE reaction produced a single fragment, which was then cloned and sequenced (Figure 2D; 100 and 10 clones for the 3′ and 5′ end determination, respectively). All clones generated from 5′ RACE used to determine the 5′-terminal sequence of the RNA molecule(s) bound to p33 shared the same sequence that corresponded to the very 5′end of the CTV gRNA (data not shown). Remarkably, most reads obtained by sequencing of the clones produced from 3′ RACE ended between 744 to 747 nt positions (Figure 2E; Supplementary Table S2) implying that the bound RNA could be a single species.

### 3.3. CTV p33 Specifically Binds the Viral Non-Coding sgRNA LMT1

**Figure 3:**
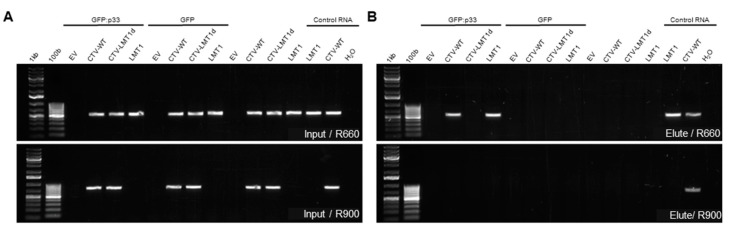
Non-coding sgRNA LMT1 produced from CTV binds to the p33 protein. The images show RT-PCR products analyzed by agarose gel electrophoresis. Each panel shows RT-PCR products using the total RNA extracted from the supernatant of the lysed homogenate of the leaf samples (Input, (**A**)) and the total RNA extracted from GFP-Trap beads at the completion of the RIP assay (Elute, (**B**)). The two sets of primers (R660 and R900) used to differentiate the shorter LMT1 RNA from the longer genomic RNA are described in Results. Control RNA samples are total RNA extracted from the leaves of *N. benthamiana* agroinfiltrated with a single construct as labeled. Two different molecular weight marker sets were used (1 kb and 100 b). 1 kb-marker was described in Figure 2. Another marker (100 b) has bands incremented by 100-bp size from 100 bp to 1.0 kb. EV, empty vector.

Among the RNA molecules produced from the CTV genome during its replication is a sgRNA termed LMT1 [11,13,14]. As we showed previously, LMT1 is a viral long non-coding RNA that is involved in counter-acting host immune response [16]. The size of LMT1 for different isolates of CTV could slightly vary [11,13,14]. For the T36 isolate used in this study, an estimated size is about 750 nt, which is similar to that of the RNA molecule found to interact with the p33 protein. To confirm that p33 indeed binds the CTV LMT1, we performed RIP in which GFP:p33 was co-expressed with a mutant of CTV, which is unable to produce the LMT1 sgRNA (CTV-LMT1d; [16]), the LMT1-producing wild type CTV that is referred here as CTV-WT, or a transiently-expressed RNA that corresponds to LMT1 (pCASS-LMT1; [16]) in the *N. benthamiana* leaves (Figure 3A). Free GFP was used as a control for each treatment. Upon immunoprecipitation using GFP-Trap^®^, the bound RNA was analyzed using RT-PCR with two sets of primers with the same forward primer (FW108). One set included a reverse primer annealing at the 660 nt position (R660) for detection of the LMT1 molecule and the viral gRNA, while the other set included a reverse primer binding at the 900 nt position (R900) that was designed to detect only gRNA. The shorter product (with R660) was amplified from the “Elute” RNA when GFP:p33 was co-expressed with either CTV-WT or transiently-expressed LMT1 (Figure 3B). No product was amplified from the same RNA template by the primer set with R900, confirming that the eluted RNA was shorter than 900 nts. On the contrary, RT-PCR using the “Elute” RNA from GFP:p33 co-expressed with the LMT1-deficient CTV-LMT1d resulted in no amplified product with either set, implying that no virus-produced RNA was bound to GFP:p33. The RNA eluted in the control treatment with free GFP yielded no amplification.

**Figure 4:**
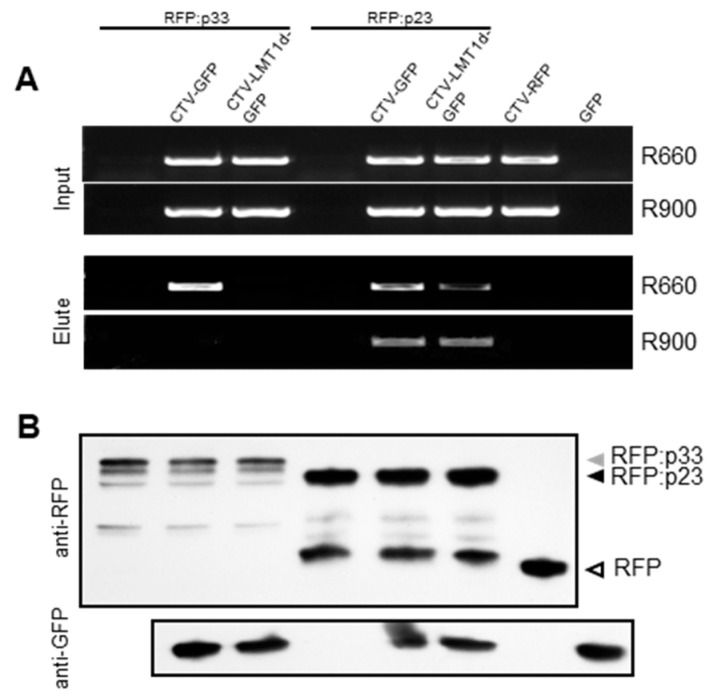
Comparison of the RNA binding properties of two CTV proteins—p33 and p23. The images show RT-PCR products and proteins in the corresponding RIP assay analyzed by agarose gel electrophoresis (**A**) or by immunoblot assay (**B**), respectively. (**A**) Each panel shows RT-PCR products generated using the total RNA extracted from the Input and Elute steps of the RIP assay (top and bottom, respectively). Two sets of primers, which included reverse primers R660 and R900, were used to differentiate the shorter LMT1 RNA from the longer genomic RNA. CTV-RFP (CTV-WT expressing free RFP) and GFP were used as controls to rule out the RFP-Trap precipitation of CTV RNA in the presence of non-tagged CTV proteins and free RFP or GFP, respectively. (**B**) Immunoblot analysis of the total protein extracts used as “Input” with an anti-GFP antibody to detect expression of free GFP produced by the CTV variants or the GFP control and an anti-RFP antibody to detect RFP-tagged proteins and free RFP produced by CTV-RFP.

Based on the results described above, p33 showed high affinity to the sgRNA LMT1, yet did not appear to bind the viral gRNA, even though the sequence of LMT1 represents a part of the 5′-terminal region of gRNA. To validate the selective binding of p33 to LMT1, we compared binding properties between p33 and the CTV p23 protein. In this experiment, the RFP-tagged p33 and p23 proteins [30] were expressed in *N. benthamiana* plants along with the GFP-tagged wild-type CTV and CTV-LMT1d (Figure 4; CTV-GFP and CTV-LMT1d-GFP, respectively). The tagged proteins were immunoprecipitated using anti-RFP antibody-linked beads (RFP-Trap^®^). As p23 was expected to bind other viral RNAs, including gRNA, in addition to LMT1, we used two sets of primers mentioned above to analyze protein-bound RNA. As we expected, both R660 and R900 yielded amplification of the respective fragments when the RNA eluted from the immunoprecipitated RFP:p23 was used as the template in the RT-PCR reactions, confirming that, besides LMT1, p23 protein is able to bind viral RNA containing regions downstream of that encoding LMT1 (Figure 4A). In contrast, RT-PCR using the “Elute” RNA in the treatment using RFP:p33 and CTV-GFP showed amplification of the shorter but not the longer fragment, which was similar to the result shown in Figure 1B and Figure 3B. No fragment was amplified from the post-RIP RNA obtained in the treatments using RFP:p33 alone or with CTV-LMT1d-GFP, indicating that no LMT1 molecule was produced, and p33 was unable to bind gRNA. We also included the wild-type CTV tagged with RFP (CTV-RFP) as a control to rule out a possibility of non-specific binding of CTV RNA to RFP. No amplification from the RNA eluted from the RFP-Trap^®^ beads incubated with the extract from the leaves infiltrated with and CTV-RFP was detected, which confirmed the absence of a non-specific interaction between LMT1 and RFP. Expression of RFP:p23, RFP:p33, free RFP, and free GFP in the respective treatments was verified by Western blot analysis using RFP- or GFP- specific antibodies, respectively (Figure 4B).

### 3.4. CTV p33 Binds to at Least Two Distinct Regions within LMT1

**Figure 5:**
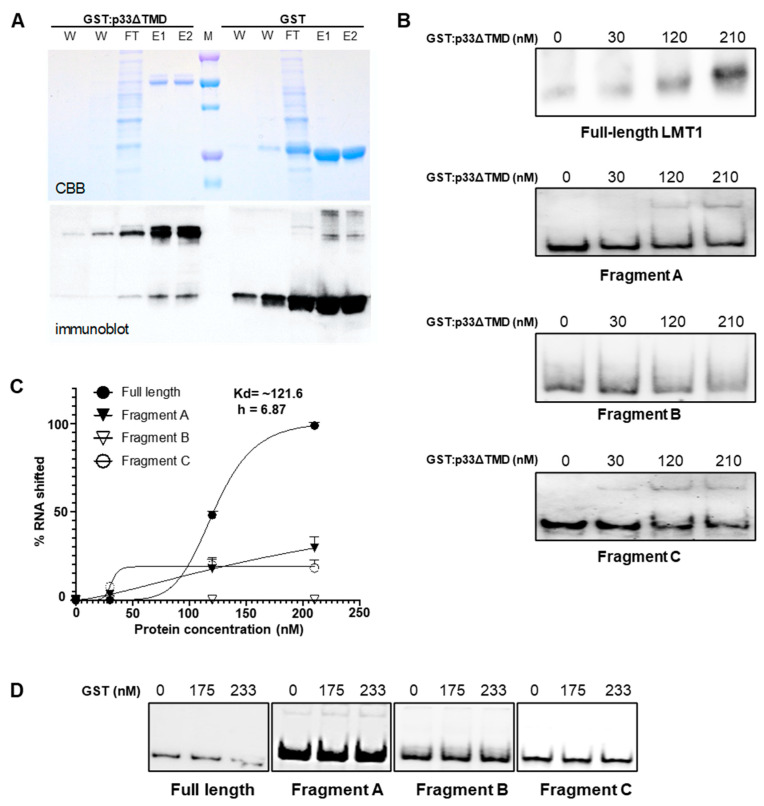
In vitro binding of purified p33 protein and LMT1 RNA or its truncated versions. (**A**) SDS-PAGE analysis of purified GST:p33ΔTMD and GST proteins followed by Coomassie Brilliant Blue (CBB) staining. Proteins were verified using an anti-GST antibody after the transfer to the PVDF membrane (immunoblot). W, wash fraction; FT, flow-through fraction; E, elution fraction. (**B**) Kinetics of p33 and LMT1 binding was analyzed using a PAGE loaded with a series of varying amounts of purified GST:p33ΔTMD with the same amount of LMT1 transcripts or LMT-1-derived fragments. (**C**) The binding kinetics between p33 and LMT-1 or LMT1-derived fragments were plotted to the Hill equation. The Kd and Hill coefficient (h) of p33 affinity to full-length LMT1 are listed. (**D**) GST alone did not bind with any of the tested RNA as shown by EMSA.

To define a region(s) of LMT1 involved in binding to the p33 protein, we conducted EMSA assays using a purified p33 protein and in vitro-transcribed RNA molecules generated from a set of constructs in which truncated fragments of the LMT1 sequence were cloned under the T7 or SP6 promoter. To purify p33, we first cloned a full-length p33 ORF into the pGEX-4T-1 expression vector fused with an upstream GST tag for protein expression in *E. coli*. However, the yield of the GST-tagged p33 purified using Glutathione Sepharose™ 4B beads was very low due to its insolubility. To circumvent this obstacle, a sequence encoding p33 lacking its C-terminal TMD was cloned into the expression vector, and GST:p33ΔTMD was expressed and successfully purified using the procedures described above and in the Section 2 (Figure 5A). Next, we confirmed that the p33 protein with truncated TMD was still able to bind LMT1 in the RIP assay using the extracts from *N. benthamiana* leaves co-expressing GFP:p33ΔTMD [26] with either the CTV-WT or CTV-LMT1d followed by RT-PCR with the R660 and R900 primer sets as described above (Supplementary Figure S3). Similar to what was observed with the full-length p33 protein, a primer set with R660 but not the one with R900 amplified a product corresponding to LMT1 when the “Elute” RNA in the treatment with GFP:p33ΔTMD and CTV-WT was used as the template for RT-PCR. No amplification occurred in the treatment with CTV-LMT1d. This result confirmed that truncation of TMD did not affect the ability of p33 protein in binding sgRNA LMT1, thus, supporting the employment of p33ΔTMD in the EMSA assay.

To examine the interaction, increasing amounts of purified GST:p33ΔTMD—as well as the GST protein alone—were incubated with in vitro-transcribed LMT1 RNA, separated on a non-denaturing gel, and transferred onto a nylon membrane. The membrane was hybridized with a DIG-labelled 5′ CTV probe described above (see also Figure 1A) to detect LMT1. A shift in mobility was not observed until GST:p33ΔTMD protein concentration was increased to 120 nM (Figure 5B; full-length LMT1). p33 reached saturated binding with 1 µM full-length LMT1 at a concentration of 210 nM. After fitting to the Hill equation, the affinity and the Hill coefficient were estimated as 121.6 nM and 6.87, respectively (Figure 5C; Full length). This in vitro binding confirmed that p33ΔTMD binds LMT1 directly and does not need any host or virus factors as intermediates in binding. Furthermore, as the Hill coefficient reflects the cooperativity of protein and the target, the high Hill coefficient reading reflects positive cooperative binding (i.e., multiple binding sites on LMT1).

To identify the regions of LMT1 responsible for binding to p33, we first analyzed the LMT1 sequence using the mFOLD program, which predicted that the LMT1 secondary structure could be quite complex and have a number of clustered stem-loop structures [40] (Supplementary Figure S2). For further mapping of the binding region(s), we chose three fragments encompassing nt positions 201–400, 401–600, and 601–780 (fragments A, B, and C, respectively). We did not include the fragment encompassing nt positions 1–200 because this region alone cannot form a structure similar to the full-length RNA. As we determined earlier, the 3′ end of LMT1 maps to the nt position 747 (see Figure 2). Based on the mFOLD prediction showing that the structure of the 5′-terminal 747-nt fragment in the CTV genome representing LMT1 (Supplementary Figure S2) is highly similar to that of the 5′-terminal 780 nt fragment, we chose a slightly longer length for the fragment C to keep the length of all fragments used in the EMSA analysis comparable and, consequently, a longer length (780 nts) for a full-length RNA. Importantly, a prominent cluster of stem-loop structures in fragment C located between nt positions 615 and 728 was preserved in both the 601–747 nt fragment (Supplementary Figure S2) and that encompassing nt positions 601–780 (data not shown). Sequences of fragments A, B, and C were cloned into a vector plasmid for in vitro transcription of RNA molecules to be used in EMSA analysis. A shift in mobility was detected with fragments A and C, but not with B (Figure 5B). Furthermore, it appeared that the p33 did not reach saturated binding of the A and C fragments as compared to that of the full-length LMT1 (Figure 5B,C). Therefore, it appears that cooperative binding accounts for the full binding affinity of the full-length transcript. We could not estimate the affinities to fragments A and C with confidence because we couldn’t observe saturated shifting. Notably, GST alone did not bind to either full-length LMT1 or fragments (Figure 5D). These results suggested that there were at least two distinct p33 binding sites in LMT1.

## 4. Discussion

CTV is one of the most intricate viruses with a complex biology. Among the peculiar features of CTV is the production of a viral lncRNA LMT1. The 5′-terminal sgRNA LMT1 was discovered more than two decades ago, however, for years, its role in virus biology has remained unknown [41]. Recently, we showed that LMT1 is involved in counter-acting host defense response to CTV infection [16]. The p33 protein represents another remarkable trait of CTV. This unique protein is required for virus infection of a few selective citrus hosts yet it is dispensable for CTV infection of many other varieties of citrus [20,22]. With that, p33 could be regarded as the “champion” among other CTV proteins for various functions it performs in the virus infection cycle, which includes the involvement of p33 in viral superinfection exclusion, interaction with host immunity, and translocation in certain hosts (reviewed in [8]). Here, we show that p33 binds LMT1 with high affinity. We also demonstrate that at least two regions within LMT1 participate in the p33-LMT1 interaction. On the other hand, the p33 protein was not found to bind other CTV RNAs, suggesting that the observed interaction between p33 and LMT1 is highly specific.

Previously, many plant viral proteins were shown to bind RNA. A large number of these proteins are MPs, which interact with RNA in a sequence-non-specific manner, so they bind viral and non-viral RNA molecules with similar affinity (reviewed in [31]). Our earlier studies demonstrated that p33 shares multiple characteristics with MPs encoded in the genomes of other plant viruses [26,28,30]. In that respect, selective binding of the CTV p33 protein to a specific viral RNA species shown in this study is noteworthy and suggests that the RNA-binding property of p33 could be associated with a distinct function. In contrast to MPs, viral proteins that participate in virus replication by recruiting viral RNAs into replication complexes recognize and bind specific sequences within these RNA molecules. Examples of such proteins include *Tomato bushy stunt virus* (TBSV) replicase protein p33 that binds to an internal replication element of TBSV gRNA [42], RCNMV auxiliary replication protein p27 that recognizes a Y-shaped element in the 3′ untranslated region (UTR) of RNA2, which mediates its recruitment [43,44], and *Brome mosaic virus* (BMV) protein 1a that specifically interacts with the box B motifs in the BMV gRNAs [45,46]. For the TBSV p33 protein, it was shown that the specificity of RNA binding was dependent on the ability of p33 to form multimeric complexes [42]. This finding is in the agreement with a general observation that dimerization plays an important role in the specificity of RNA recognition as it provides multiple sites for RNA binding and strengthens the affinity of the protein to the RNA [47]. Interestingly, earlier, we demonstrated that CTV p33 also possesses a self-interaction ability [48], and this study showed a cooperative binding affinity of p33 to its RNA target.

The results of this work reporting binding of the CTV p33 protein, which is dispensable for virus replication, assembly, as well as cell-to-cell movement, to a viral lncRNA LMT1 stands out from known cases of plant viral protein-RNA interactions. LncRNAs were identified in a wide range of organisms, including prokaryotes, eukaryotes, and viruses, and represent RNA molecules with lengths more than 200 nts and no apparent protein-coding capacity [49]. LncRNAs have been reported to be involved in numerous biological processes such as transcription regulation, microRNA suppression, cellular differentiation, development, and regulation of biotic and abiotic stresses [50]. Importantly, lncRNAs were often found interacting with RNA-binding proteins and acting as scaffolds for the formation of RNA-protein complexes or altering the activity and/or localization of the respective proteins [51,52]. Furthermore, lncRNAs play important roles in virus-host interactions. Many plant and animal lncRNAs are induced as either pro-viral or anti-viral factors upon viral infections [51,52]. Remarkably, several viruses have been shown to produce lncRNAs themselves that are often multifunctional. One example of such viral lncRNA is a subgenomic flavivirus RNA (sfRNA) derived from a 3′ UTR of a flavivirus genome. sfRNAs produced by different flaviviruses, including West Nile and dengue viruses, were found to suppress small-interfering RNA- and microRNA-induced RNA interference pathways by acting as competitive substrates for Dicer [53,54], bind and inhibit TRIM25, a protein vital for the activation of host antiviral response activation, or interact with host RNA-binding proteins that regulate interferon-stimulated genes [55,56]. Adding to the multifunctionality of sfRNAs, Zika virus sfRNA was reported to sequester an antiviral DEAD/H-box helicase ME31B, promoting virus replication and virion production to aid transmission by mosquitoes [57]. Another example is the polyadenylated nuclear (PAN) RNA produced by Kaposi’s sarcoma-associated Virus (KSHV), a DNA herpesvirus. PAN RNA interacts with several host RNA-binding proteins to regulate viral and host gene expression [58,59,60], promote reactivation of KHSV by sequestering viral latency protein [61], and modulate immunity [62].

Plant virus lncRNAs have not received the same amount of attention as their animal virus counterparts, and our understanding of their functions remains limited. *Beet necrotic yellow vein virus* produces ncRNA3, a 500-nt long lncRNA with unknown function [63]. Another example is the 400-nt SRf1 produced by RCNMV. SRf1 originates from the 3′ UTR of RNA1 and suppresses cap-independent and cap-dependent translation as well as consequently represses the synthesis of the negative strand of RCNMV RNA by limiting the production of the replicase protein [64]. Unlike CTV LMT1, which is produced by the termination of RNA synthesis at a controller element on the negative strand complementary to gRNA [11,15], these two lncRNAs are products of degradation of gRNAs [63,64].

Although viral lncRNAs often bind the host proteins, fewer cases of the interactions between the former RNAs with proteins produced by the virus are known. One of them is the binding of KSHV PAN RNA to several KSHV proteins, which mediates regulation of the lytic reactivation and, overall, viral gene expression [65]. As for plant virus lncRNAs, to the best of our knowledge, binding of the CTV p33 protein to the CTV lncRNA LMT1 is the first report of a plant viral protein binding to a lncRNA produced by the same virus.

According to our recent study, the lack of LMT1 production by a CTV mutant reduced virus invasiveness and systemic movement in the CTV experimental host *N. benthamiana* and precluded citrus infection [16]. Furthermore, virus-driven or ectopic expression of LMT1 was found to modulate plant antiviral response by suppressing salicylic acid signaling and upregulating alternative oxidase, which limits Reactive oxygen species (ROS) production. Interestingly, the p33 protein is also involved in the virus interplay with host immunity. However, in contrast to LMT1, it triggers host defense against CTV infection, including the increase in the accumulation of ROS [25,27]. It is possible that interaction between these viral factors modulates host immune response to CTV infection and allows the virus to persist in the long-lived woody citrus host for the whole tree life, while reaching sufficient titers to be transmitted by the aphid vector to a new host.

## Supplementary Materials

The following are available online at https://www.mdpi.com/article/10.3390/v13112129/s1, Figure S1: RNA binding motif of the p33 protein, Figure S2: Schematic representation of the LMT1 secondary structure prediction generated by mFOLD, Figure S3: LMT1 binding of the p33 protein lacking its transmembrane domain, Table S1: List of primers used in this study, Table S2: Sequence of clones produced from 3′ RACE. All authors have read and agreed to the published version of the manuscript.
