# Supplementary material for: The Intriguing Conundrum of a Nonconserved Multifunctional Protein of Citrus Tristeza Virus That Interacts with a Viral Long Non-Coding RNA
Source: Viruses. 2021 Oct 22;13(11):2129. doi: 10.3390/v13112129 (PMC8625556; doi:10.3390/v13112129)
Supplement: Supplementary file 1 [file viruses-13-02129-s001.zip › viruses-1414750-SI.pdf]

Supplementary Materials

10 20 30 aa

MFAFASESQDLLLEEKVF**RRRTYHRK**YFGDVVKDFTID

Supplementary Figure S1. RNA binding motif of the p33 protein. The N-terminal amino acid sequence of the p33 protein from the T36 isolate of CTV is shown. A predicted canonical arginine-rich. RNA-binding motif is marked in bold characters.

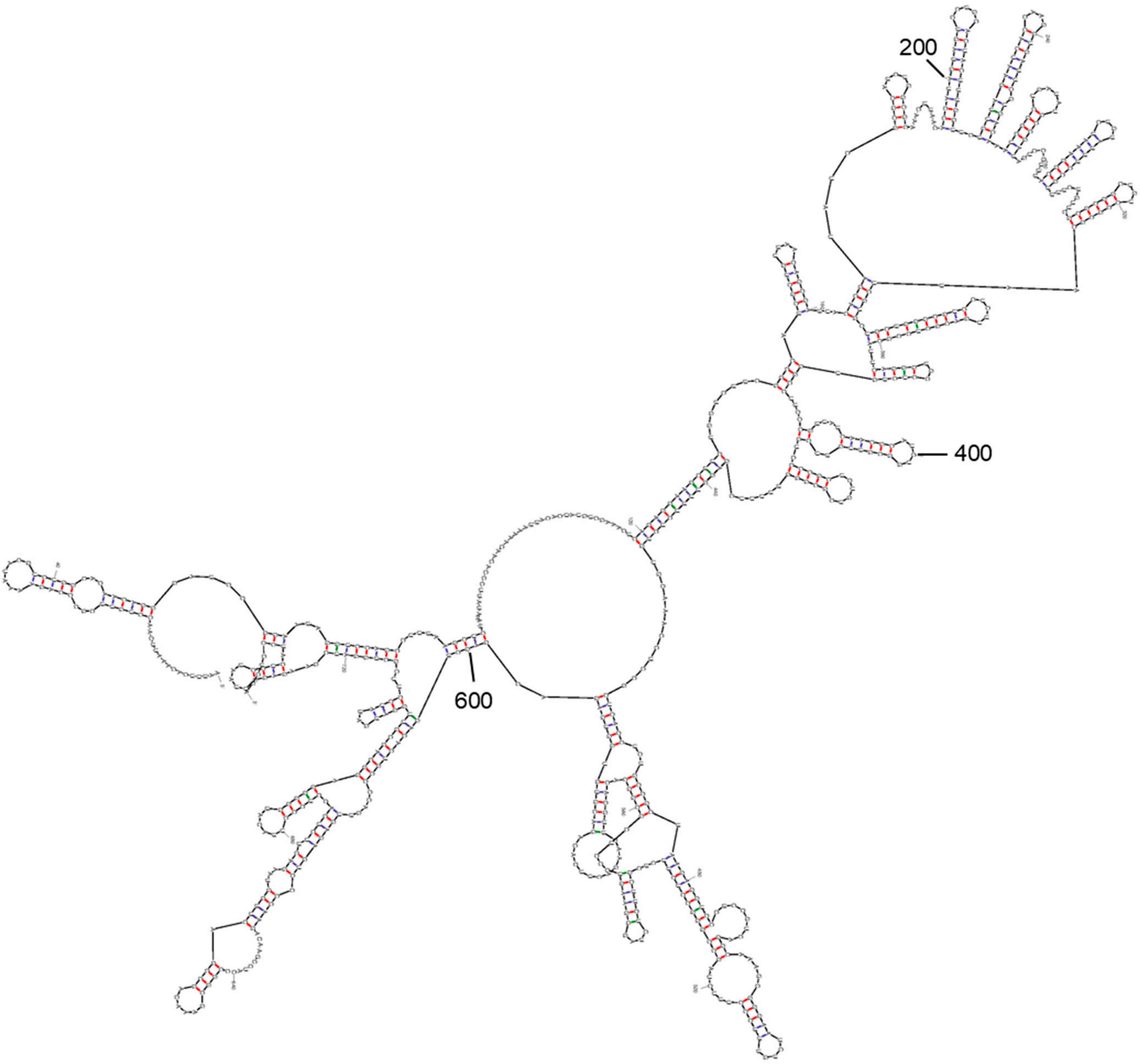

Supplementary Figure S2. Schematic representation of the LMT1 secondary structure prediction generated by mFOLD. The nucleotide positions in the LMT1 sequence are shown.

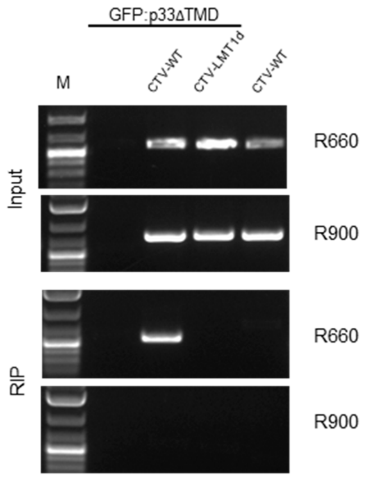

Supplementary Figure S3. LMT1 binding of the p33 protein lacking its transmembrane domain. The images show RT-PCR products analyzed by agarose gel electrophoresis following a RIP assay using a GFP-tagged p33OTMD. Panels shows RT-PCR products using the total RNA extract (Input) or the RNA extract from the pelleted agarose beads after RIP. Two sets of primers used for the identification of the bound RNA size were described in Fig. 3. The molecular marker (M) on the far-left lane of each agarose gel was described in Fig. 2.

Supplementary Table S1. List of primers used in this study.

Primer Name	Sequence (5′ to 3′)	ReferredFig. No.
FW108	ATGTCGAAACTCAGAGGAAGCTTC	1, 2, 3, 4
R540	GCGAGAAGAGGCCCGGCTAGAAAGGG	1, 2
P33-FW	CAGTGAATTCATGTTTGCCTTCGCGAGCGAAAGC	1, 5
P33-RV	TCATATAAATATAATGGCTAATAAACCG	1
P23-FW	CAGTGAATTCATGGATAATACTAGCGGACAAAC	1
P23-RV	TCAGATGAAGTGGTGTTCACGGAG	1
R600	CTCGTCTCACCACACGTCTG	2
R660	CAAGTCGGCTGTTTCGCCGGG	2, 3, 4
R700	CGCTGCCTCCCCCATGTAAGGG	2
R750	CCATCGAGGGATGAGGATTAA	2
R800	CCAAGCCGTTGCAGGCATGG	2
R850	CGCGTGATAGTCAAGGCGGC	2
R900	GACGTACCGTACGTGTTTCTCC	2, 3, 4
R1050	CGCGTGAGAAGATCTCACGAACG	2
R1550	GGTTAGGAATAGATCGCAGGTCG	2
R2050	GCCACGCTTTACAAACACGTG	2
GSP1	GATTACGCCAAGCTTGGCTGTTTCGCCGGGTAGGCTTGTCTTGGG	2
GSP2	GATTACGCCAAGCTTGAGGAAGCTTCTGGTCTTCGGCCATCGCTG	2
P33ΔTMD-RV	ATTTGCGGCCGCTTAGACCCGGCGTATTAATG	5
LMT FW 1	AATTTCACAAATTCAACCTG	5
LMT FW 200	GTAGTCCTGCACTACTTTG	5
LMT FW 400	TGGCGTTGTTCGTCCCCG	5
LMT FW 600	CTGACACTGCCCCAGTTG	5
LMT RV 400	GATGCGTTGATAACGGGAAG	5
LMT RV 600 *	CTCGTCTCACCACACGTCTG	5
LMT RV 780	TTCCAAAAGTGATGGGCAC	5
M13FW	CGCCAGGGTTTTCCCAGTCACGAC	
M13RV	CAGGAAACAGCTATGAC	

* LMT RV 600 is identical to R600.

Supplementary Table S2. Sequence of clones produced from 3′ RACE. Table shows the sequencing results for 90 clean reads out of 100 clones rescued from the excised amplified product by 3′(+) RACE described in Fig. 2D. The "Ending nt" column shows the nt position of the last nt read from the clone on CTV genome. The frequency of each ending nt was shown in Fig. 2E.

Clone No.	5′ to 3′ Sequence at 3′ terminus	Ending nt
7	TTGGATGATATCCTTCGCTGGATCGAAGAGGTTAATCCTCATCCCTCGA	747
8	TTGGATGATATCCTTCGCTGGATCGAAGAGGTTAATCCTCATCCCTCG	746
9	TTGGATGATATCCTTCGCTGGATCGAAGAGGTTAATCCTCATCCCTCG	746
10	TTGGATGATATCCTTCGCTGGATCGAAGAGGTTAATCCTCATCCCT	744
12	TTGGATGATATCCTTCGCTGGATCGAAGAGGTTAATCCTCATCCCT	744
13	TTGGATGATATCCTTCGCTGGATCGAAGAGGTTAATCCTCATCCCTC	745
14	TTGGATGATATCCTTCGCTGGATCGAAGAGGTTAATCCTCATCCCTCGA	747
16	TTGGATGATATCCTTCGCTGGATCCAAGAGGTTAATCCTC	738
17	TTGGATGATATCCTTCGCTGGATCGAAGAGGTTAATCCTCATCCCTCGA	747
20	TTGGATGATATCCTTCGCTGGATCGAAGAGGTTAATCCTCATCCCT	744
21	TTGGATGATATCCTTCGCTGGATCGAAGAGGTTAATCCTCATCCCTCGA	747
22	TTGGATGATATCCTTCGCTGGATCGAAGAGGTTAATCCTCATCCCTCGA	747
23	TGGAGGATATCCTTCGCTGGATGGAAGAGGTTAATCCTCATCCCTCGC	747
24	TTGGATGATATCCTTCGCTGGATCGAAGAGGTTAATCCTCATCCCTCGA	747
27	TTGGATGATATCCTTCGCTGGATCGAAGAGGTTAATCCTCATCCCTCGA	747
28	TTGGATGATATCCTTCGCTGGATCGAAGAGGTTAATCCTCATCCCTCG	746
29	TTGGATGATATCCTTCGCTGGATCGAAGAGGTTAATCCTCATCCCTC	745
30	TTGGATGATATCCTTCGCTGGATCGAAGAGGTTAATCCTCATCCCTCGA	747
11	TTGGATGATATCCTTCGCTGGATCGAAGAGGTTAATCCTCATCCCTCGA	747
15	TTGGAGTAATATCCTTTNNTGGATGGAGGAGGNTGATCATAATCCTTCGA	747
18	TTGGATGATATCCTTCGCTGGATCGAAGAGGTTAATCCTCATCCCTCGA	747
19	TTGGATGATATCCTTCGCTGGATCGAAGAGGTTAATCCTCATCCCTCGA	747
25	TTGGATGATATCCTTCGCTGGATCGAAGAGGTTAATCCTCATCCCTCGA	747
26	TTGGATGATATCCTTCGCTGGATCGAAGAGGTTAATCCTCATCCCTCGA	747
31	TTGGATGATATCCTTCGCTGGATCGAAGAGGTTAATCCTCATCCCTCGA	747
32	TTGGATGATATCCTTCGCTGGATCGAAGAGGTTAATCCTCATCCCTC	745
33	TTGGATGATATCCTTCGCTGGATCGAAGAGGTTAATCCTCATCCCTCG	746
34	TTGGATGATATCCTTCGCTGGATCGAAGAGGTTAATCCTCATCCCTCGA	747
35	TTGGATGATATCCTTCGCTGGATCGAAGAGGTTAATCCTCATCCCTCGA	747
36	TTGGATGATATCCTTCGCTGGATCGAAGAGGTTAATCCTCATCCCT	744
37	TTGGATGATATCCTTCGCTGGATCGAAGAGGTTAATCCTCATCCCT	744
38	TTGGATGATATCCTTCGCTGGATCGAAGAGGTTAATCCTCATCCCTC	745
39	TTGGATGATATCCTTCGCTGGATCGAAGAGGTTAATCCTCATCCCTCG	746
40	TTGGATGATATCCTTCGCTGGATCGAAGAGGTTAATCCTCATCCCTCG	746
41	TTGGATGATATCCTTCGCTGGATCGAAGAGGTTAATCCTCATCCCT	744
42	TTGGATGATATCCTTCGCTGGATCGAAGAGGTTAATCCTCATCCCTCGA	747
43	TTGGATGATATCCTTCGCTGGATCGAAGAGGTTAATCCTCATCCCT	744
44	TTGGATGATATCCTTCGCTGGATCGAAGAGGTTAATCCTCATCCCTCGA	747
45	TTGGATGATATCCTTCGCTGGATCGAAGAGGTTAATCCTCATCCCTCGA	747
46	TTGGATGATATCCTTCGCTGGATCGAAGAGGTTAATCCTCATCCCTCGA	747
47	TTGGATGATATCCTTCGCTGGATCGAAGAGGTTAATCCTCATCCCT	744
48	TTGGATGATATCCTTCGCTGGATGGAAGAGGTTAATCCTCATCCCTCGA	747
49	TTGGATGATATCCTTCGCTGGATCGAAGAGGTTAATCCTCATCCCTCGA	747
50	TTGGATGATATCCTTCGCTGGATCGAAGAGGTTAATCCTCATCCCT	744
51	TTGGATGATATCCTTCGCTGGATCGAAGAGGTTAATCCTCATCCCTCG	746
52	TTGGATGATATCCTTCGCTGGATCGAAGAGGTTAATCCTCATCCCTCGA	747
53	TTGGATGATATCCTTCGCTGGATCGAAGAGGTTAATCCTC	738
54	TTGGATGATATCCTTCGCTGGATCGAAGAGGTTAATCCTCATCCCTCGA	747

55	TTGGATGATATCCTTCGCTGGATCGAAGAGGTTAATCCTCNTCCCT	744
56	TTGGATGATATCCTTCGCTGGATCGAAGAGGTTAATCCTCATCCCTCGA	747
57	TTGGATGATATCCTTCGCTGGATCGAAGAGGTTAATCCTCATCCCTCG	746
58	TTGGATGATATCCTTCGCTGGATCGAAGAGGTTAATCCTCATCCCT	744
59	TTGGATGATATCCTTCGCTGGATCGAAGAGGTTAATCCTCATCCCT	744
60	TTGGATGATATCCTTCGCTGGATCGAAGAGGTTAATCCTCATCCCTCGA	747
62	TTGGATGATATCCTTCGCTGGATCGAAGAGGTTAATCCTCATCCCT	744
63	TTGGATGATATCCTTCGCTGGATCGAAGAGGTTAATCCTCATCCCT	744
64	TTGGATGATATCCTTCGCTGGATCGAAGAGGTTAATCCTCATCCCTCGA	747
65	TTGGATGATATCCTTCGCTGGATCGAAGAGGTTAATCCTCATCCCTCGA	747
67	TTGGATGATATCCTTCGCTGGATCGAAGAGGTTAATCCTCATCCCTCGA	747
68	TTGGATGATATCCTTCGCTGGATCGAAGAGGTTAATCCTCATCCCTCGA	747
70	TTGGATGATATCCTTCGCTGGATCGAAGAGGTTAATCCTCATCCCTC	745
71	TTGGATGATATCCTTCGCTGGATCGAAGAGGTTAATCCTCATCCCTCGA	747
73	TTGGATGATATCCTTCGCTGGATCGAAGAGGTTAATCCTCATCCCTCGA	747
75	TTGGATGATATCCTTCGCTGGATCGAAGAGGTTAATCCTCNNCCCT	744
76	TTGGATGATATCCTTCGCTGGATCGAAGAGGTTAATCCTCATCCCTCGA	747
77	TTGGATGATATCCTTCGCTGGATCGAAGAGGTTAATCCTCATCCCTCG	746
78	TTGGATGATATCCTTCGCTGGATCGAAGAGGTTAATCCTCATCCCTCGA	747
79	TTGGATGATATCCTTCGCTGGATCGAAGAGGTTAATCCTCATCCCTCGA	747
80	TTGGATGATATCCTTCGCTGGATCGAAGAGGTTAATCCTCATCCCTCGA	747
82	TTGGATGATATCCTTCGCTGGATCGAAGAGGTTAATCCTCATCCCTC	745
83	TTGGATGATATCCTTCGCTGGATCGAAGAGGTTAATCCTCATCCCTCGA	747
84	TTGGATGATATCCTTCGCTGGATCGAAGAGGTTAATCCTCATCCCTCG	746
85	TTGGATGATATCCTTCGCTGGATCGAAGAGGTTAATCCTCATCCCTCG	746
86	TTGGATGATATCCTTCGCTGGATCGAAGAGGTTAATCCTCATCCCTCG	746
87	TTGGATGATATCCTTCGCTGGATCGAAGAGGTTAATCCTCATCCCTCGA	747
88	TTGGATGATATCCTTCGCTGGATCGAAGAGGTTAATCCTCATCCCT	744
89	TTGGATGATATCCTTCGCTGGATCGAAGAGGTTAATCCTCATCCCTCGA	747
90	TTGGATGATATCCTTCGCTGGATCGAAGAGGTTAATCCTCATCCCTCG	746
91	TTGGATGATATCCTTCGCTGGATCGAAGAGGTTAATCCTCNTCCCT	744
92	TTGGATGATATCCTTCGCTGGATCGAAGAGGTTAATCCTCATCCCTCG	746
93	TTGGATGATATCCTTCGCTGGATCGAAGAGGTTAATCCTCATCCCT	744
94	TTGGATGATATCCTTCGCTGGATCGAAGAGGTTAATCCTCATCCCTCG	746
95	TTGGATGATATCCTTCGCTGGATCGAAGAGGTTAATCCTCATCCCTCGA	747
96	TTGGATGATATCCTTCGCTGGATCGAAGAGGTTAATCCTCATCCCTCG	746
97	TTGGATGATATCCTTCGCTGGATCGAAGAGGTTAATCCTCATCCCT	744
98	TTGGATGATATCCTTCGCTGGATCGAAGAGGCTAATCCTCATCCCTCGA	747
99	TTGGATGATATCCTTCGCTGGATCGAAGAGGTTAATCCTCATCCCTCGA	747
100	TTGGATGATATCCTTCGCTGGATCGAAGAGGTTAATCCTCATCCCTCGA	747
101	TTGGATGATATCCTTCGCTGGATCGAAGAGGTTAATCCTCATCCCTCGA	747
102	TTGGATGATATCCTTCGCTGGATCGAAGAGGTTAATCCTCNNCCCT	744
